# Mapping of intracellular pH in the in vivo rodent heart using hyperpolarized [1‐13C]pyruvate

**DOI:** 10.1002/mrm.26260

**Published:** 2016-05-13

**Authors:** Angus Z. Lau, Jack J. Miller, Damian J. Tyler

**Affiliations:** ^1^ Division of Cardiovascular Medicine, Radcliffe Department of MedicineUniversity of OxfordUK; ^2^ Department of PhysiologyAnatomy, and Genetics, University of OxfordUK; ^3^ Department of Physics, Clarendon LaboratoryUniversity of OxfordUK

**Keywords:** hyperpolarization, pH, Cardiac, ^13^C

## Abstract

**Purpose:**

To demonstrate the feasibility of mapping intracellular pH within the in vivo rodent heart. Alterations in cardiac acid‐base balance can lead to acute contractile depression and alterations in Ca^2+^ signaling. The transient reduction in adenosine triphosphate (ATP) consumption and cardiac contractility may be initially beneficial; however, sustained pH changes can be maladaptive, leading to myocardial damage and electrical arrhythmias.

**Methods:**

Spectrally selective radiofrequency (RF) pulses were used to excite the ${\text{HCO}}_{3}^{-}$ and CO_2_ resonances individually while preserving signal from the injected hyperpolarized [1‐^13^C]pyruvate. The large flip angle pulses were placed within a three‐dimensional (3D) imaging acquisition, which exploited CA‐mediated label exchange between ${\text{HCO}}_{3}^{-}$ and CO_2_. Images at 4.5 × 4.5 × 5 mm^3^ resolution were obtained in the in vivo rodent heart. The technique was evaluated in healthy rodents scanned at baseline and during high cardiac workload induced by dobutamine infusion.

**Results:**

The intracellular pH was measured to be 7.15 ± 0.04 at baseline, and decreased to 6.90 ± 0.06 following 15 min of continuous β‐adrenergic stimulation.

**Conclusions:**

Volumetric maps of intracellular pH can be obtained following an injection of hyperpolarized [1‐^13^C]pyruvate. The new method is anticipated to enable assessment of stress‐inducible ischemia and potential ventricular arrythmogenic substrates within the ischemic heart. Magn Reson Med 77:1810–1817, 2017. © 2016 The Authors Magnetic Resonance in Medicine published by Wiley Periodicals, Inc. on behalf of International Society for Magnetic Resonance in Medicine. This is an open access article under the terms of the Creative Commons Attribution License, which permits use, distribution and reproduction in any medium, provided the original work is properly cited.

## INTRODUCTION

The acid‐base balance within the normal functioning heart is tightly regulated 1. Changes in intracellular pH can lead to acute contractile depression, alterations in Ca^2+^ signaling, and electrical arrhythmias 2. For example, myocardial ischemia and poor coronary perfusion result in increased anaerobic metabolism and production of lactic acid. The ischemic heart benefits from this initial reduction in cardiac contraction, by preserving adenosine triphosphate (ATP) levels for ion channel homeostasis. Sustained ischemia, however, is maladaptive, as inhibition in Na^+^/K^+^‐ATPase activity and Na^+^/Ca^2+^ exchange lead to calcium overload within the heart.

Few methods exist to noninvasively characterize the intracellular pH (pH_i_) within the body 3. ^31^P magnetic resonance spectroscopy (MRS) has long been a gold standard for pH_i_ measurement within the isolated perfused heart 4, 5. This measurement is based on the pH‐dependent chemical shift of the inorganic phosphate (P_i_) resonance, but is practically limited to ex vivo studies due to 2,3‐diphosphoglycerate (2,3‐DPG) found in ventricular blood, which contaminates the myocardial P_i_ signal 6, 7. In vivo measurement using ^31^P‐MRS of the intracellular pH across the whole heart is possible by a saturation transfer experiment designed to separate the signal contributions from the intracellular, pH‐sensitive, P_i_, and the extracellular 2,3‐DPG 8, but low sensitivity limits this approach to whole‐heart assessment with long scan times. Numerous alternative methods exist for monitoring the pH using magnetic resonance, including spectroscopic probes using ^1^H, ^31^P, and ^19^F MRS 9, 10, 11, 12. The sensitivity of these techniques is inherently low, and the resulting chemical shifts are typically small and difficult to resolve at clinical field strengths. The pH‐dependent magnetization transfer between bulk water protons and the endogenous protein amide pool (chemical exchange saturation transfer (CEST)) offers promise, but requires an accurate determination of probe concentration, which is challenging to obtain in vivo 13, 14.

An attractive option for noninvasive pH_i_ measurement is to exploit the CO_2_/ ${\text{HCO}}_{3}^{-}$ buffering system of the body. These compounds exist in a pH‐dependent steady state within cardiomyocytes, mediated by a rapid exchange catalyzed by carbonic anhydrase (CA) enzymes. Specifically, ^13^C‐MRS of CO_2_ and ${\text{HCO}}_{3}^{-}$ concentrations within the heart would enable a ratiometric calculation of pH via the Henderson‐Hasselbalch formula. Unfortunately, this is infeasible using conventional (thermal equilibrium) ^13^C magnetic resonance, because of limited ^13^C natural abundance and the inherently low sensitivity of the MR experiment.

Recent developments in hyperpolarized magnetic resonance using the dissolution dynamic nuclear polarization (DNP) method provide transient signal increases in ^13^C‐labeled substrates relative to their thermal equilibrium polarization in excess of 10000‐fold 15. This molecular imaging modality enables imaging of in vivo metabolic changes in real time, within the initial minute of injection into the body. The injection of hyperpolarized [1‐^13^C]pyruvate, for example, provides key biochemical information regarding tricarboxylic acid (TCA) cycle flux as well as anaerobic metabolism via observation of [1‐^13^C]lactate 16, 17, 18. Injection of hyperpolarized ^13^C‐bicarbonate has previously been used to allow the noninvasive in vivo mapping of extracellular pH by exploiting CA‐mediated exchange within the blood and extracellular space 19. This approach has been used to map the acidic extracellular environment within tumors, which is correlated with prognosis and response to treatment. Developments have been made to extensively characterize and optimize extracellular pH measurements using ^13^C‐bicarbonate 20, 21. Other approaches to generating ^13^C‐bicarbonate have also been investigated, using base‐catalyzed hydrolysis in conjunction with biocompatible ^13^C‐enriched carbonates to reduce toxicity concerns from the presence of Cs^+^ 22. An interesting hyperpolarized substrate for extracellular pH measurements has been the application of the Good's buffer N‐(2‐acetamido)‐2‐aminoethanesulfonic acid (ACES), which exhibits a pH‐dependent chemical shift 23. The production of acetyl‐CoA from hyperpolarized [1‐^13^C]pyruvate by pyruvate dehydrogenase (PDH) involves mitochondrial decarboxylation as an initial step before entry into the TCA cycle. This results in the production of intracellular hyperpolarized ^13^CO_2_, which is vented from the mitochondrial matrix into the cytoplasm, where a cytosolic CA isoform catalyzes the rapid exchange with H^13^ ${\text{CO}}_{3}^{-}$. The ratio of these two signals can be used for determination of intracellular pH 24, 25. At physiological pH, the low ^13^CO_2_ signal (approximately 10% of myocardial H^13^ ${\text{CO}}_{3}^{-}$) is often at the limit of detection, which has up until now restricted this technique to whole‐heart assessment 18, 25. Although nonlocalized methods can be useful in physiological states, which affect the heart on a global level, they are insufficient when studying focal regions of ischemia. To use hyperpolarized ^13^C‐based intracellular pH measurements to identify focal regions of myocardial ischemia, it will be necessary to develop optimized data acquisition methods that increase the measured ^13^CO_2_ signal.

The aim of this study was to investigate the feasibility of performing in vivo pH_i_ mapping in the heart, using hyperpolarized ^13^C MRI. To this aim, we have developed an optimized data acquisition in which spectrally selective radiofrequency (RF) pulses are used to excite the ${\text{HCO}}_{3}^{-}$ and CO_2_ resonances individually while preserving signal from [1‐^13^C]pyruvate. A variable timing scheme is used to exploit CA‐mediated label exchange between ${\text{HCO}}_{3}^{-}$ and CO_2_, which enables the use of large flip angles for a 3D imaging acquisition. Images at 4.5 × 4.5 × 5 mm^3^ resolution are obtained in the in vivo rodent heart. The technique is evaluated in healthy rodents scanned at baseline and during high cardiac workload induced by dobutamine infusion. The new method is anticipated to enable new cardiovascular imaging examinations in which the severity of stress‐inducible ischemia can be evaluated.

## METHODS

All experiments were performed on an Agilent 7 Tesla (T) MRI system (Agilent, Santa Clara, California). All animal investigations conformed to Home Office Guidance on the Operation of the Animals (Scientific Procedures) Act (HMSO) of 1986, to institutional guidelines, and were approved by the University of Oxford Animal Ethics Review Committee.

### Animal Handling

Male Wistar rats (n = 5, weight = 500 ± 20 g) were scanned in this study. Two animals were used in initial pilot scans to determine the feasibility of the spectrally selective excitation scheme used for subsequent imaging studies. A further three animals were then scanned in two separate scan sessions consisting of rest and stress conditions to demonstrate the feasibility of the newly developed 3D imaging sequence, described subsequently. Anesthesia was induced at 2.5–3% isoflurane in oxygen and nitrous oxide (4:1, total of 2 L/min). Anesthesia was maintained by means of 2% isoflurane delivered to, and scavenged from, a nose cone during the experiment. A tail vein catheter was placed for intravenous injection of hyperpolarized [1‐^13^C]pyruvate. When required, a separate tail vein catheter was placed in the contralateral tail vein for delivery of dobutamine during the high cardiac workload experiments, described subsequently. Animals were then placed in a home‐built animal handling system 26. Body temperature was maintained using air heating, and a two‐lead electrocardiogram (ECG) for cardiac gating was obtained using leads placed subcutaneously into the upper forelimbs.

### Modulation of Cardiac Workload

Scans were performed in a group of three subjects (n = 3, weight 530 ± 20 g) in separate experiments under either rest or stress conditions. Cardiac workload was increased by continuous dobutamine infusion (concentration 1 mg/mL, dose 100 μg/kg/min, Hameln Pharma Plus GmbH, Germany) into the tail vein using a small animal infusion pump (Harvard Apparatus, Holliston, Massachusetts). The volume of the injection line was filled with dobutamine, and hyperpolarized [1‐^13^C] pyruvate was administered 15 min after the start of infusion through a separate tail vein catheter. Dobutamine infusion was maintained throughout the ^13^C scan. The heart rate immediately prior to hyperpolarized [1‐^13^C]pyruvate injection was 380 ± 20 bpm at rest and 450 ± 10 bpm during stress (*P* < 0.05, paired t‐test).

### Hyperpolarization

[1‐^13^C]pyruvic acid (14 M neat with 15 mM OX063 trityl radical and 0.8 mM Gd‐DOTA) was hyperpolarized in a prototype DNP hyperpolarizer at 93.979 GHz and 100 mW for 60 min 15. Dissolution was performed at a pressure of 10 bar and a temperature of ∼170 °C in 6 mL of NaOH solution resulting in a pH ∼7 solution of 80 mM [1‐^13^C]pyruvate. A total of 2 mL of hyperpolarized [1‐^13^C]pyruvate was injected over 20 s via the tail vein.

### MRI Acquisitions

^1^H images were acquired using a 72‐mm inner diameter dual‐tuned birdcage transmit/receive ^1^H/^13^C coil (Rapid Biomedical GmbH, Rimpar, Germany). ^13^C images were acquired using the same volumetric coil for RF transmission, and a two‐channel surface receive array (two 2 × 4 cm^2^ elements oriented in the left‐right direction) for signal reception (Rapid Biomedical GmbH, Rimpar, Germany). The surface coil was positioned on the anterior chest wall, and a thermally polarized 5 M ^13^C urea phantom was used to calibrate the ^13^C transmitter power before the ^13^C scans. A 180° pulse was calibrated by finding the power required to null the signal from this phantom. The $B_{1}^{+}$ for the volume ^13^C transmit coil was measured in previous phantom experiments using a double‐angle gradient echo (GRE) acquisition, and was found to have a coefficient of variation (SD/mean) of 4% over a cylindrical volume encompassing the sensitive region of the coil (diameter 40 mm, height 40 mm). A volume covering a 40‐mm slab including the heart was used for shimming using a 3D gradient‐echo automated shim routine 27. An axial stack of ECG‐gated, segmented GRE images covering the 40‐mm slab were used for anatomical reference (16 slices, pulse repetition time (TR) 3.3 ms, echo time (TE) 1.3 ms, 128 × 128 matrix, field of view (FOV) 60 × 60 mm^2^, slice thickness 2.5 mm, 8 averages, 64 segments, bandwidth (BW) 1150 Hz/pixel). ECG‐gated ^1^H cine imaging was used to assess cardiac function in a single midventricular short‐axis slice in the increased cardiac workload experiments (TR 4.6 ms, TE 1.6 ms, 128 × 128 matrix, FOV 60 × 60 mm^2^, slice thickness 1.6 mm, 8 averages, BW 1150 Hz/pixel). Function was assessed both at baseline and 5 min following the beginning of dobutamine infusion.

### RF Pulse Design

**Figure 1:**
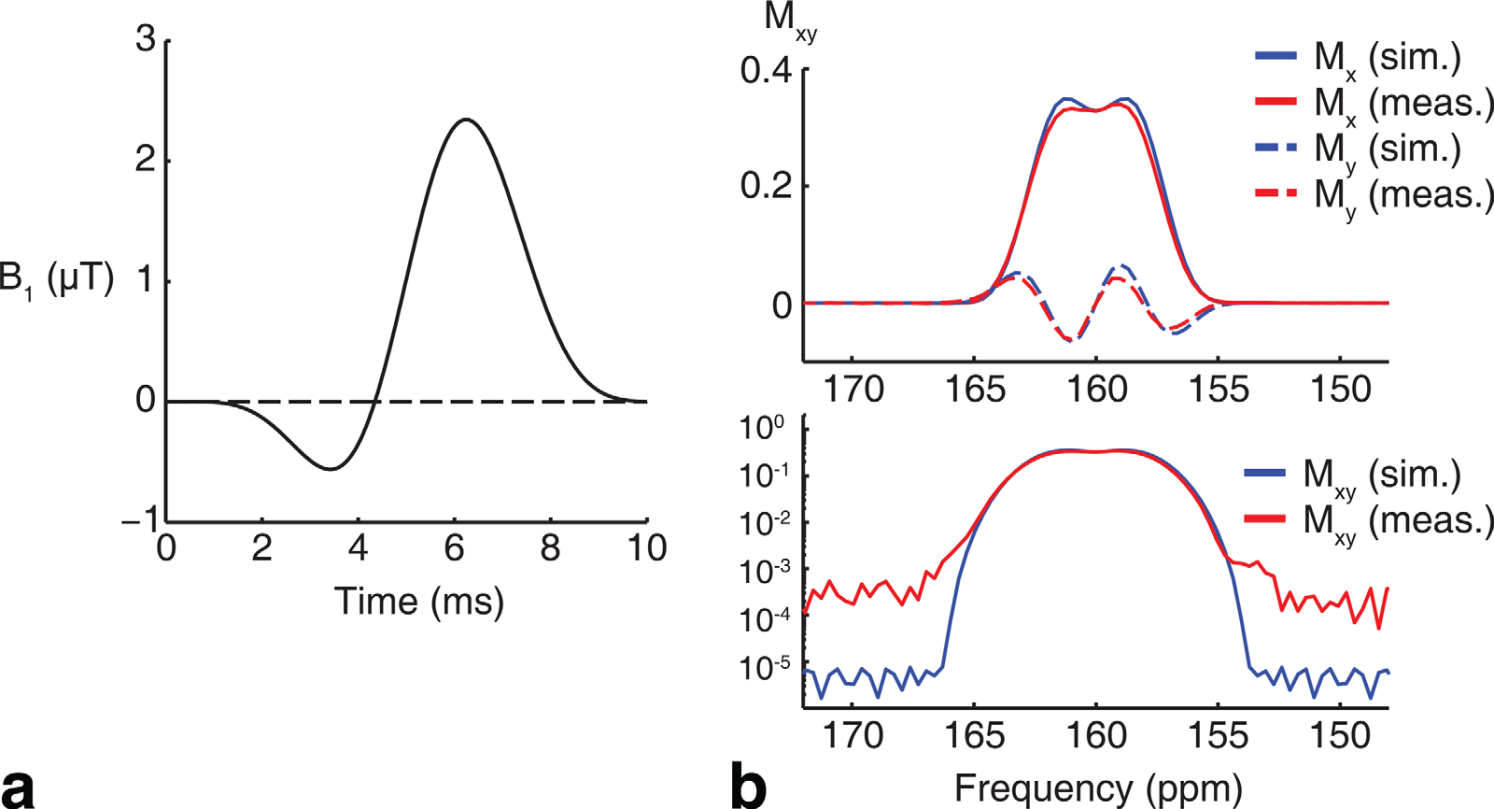
(a) Spectrally selective, minimum phase radiofrequency pulse designed to excite ${\text{HCO}}_{3}^{-}$ (160 ppm) while leaving [1‐^13^C]pyruvate (170 ppm) unexcited. (b) Experimentally measured spectral profiles using thermally polarized, 5 M ^13^C urea show good agreement with Bloch simulation (top: real and imaginary response; bottom: magnitude response) with residual errors likely due to RF amplifier nonlinearities.

Using the Shinnar Le‐Roux (SLR) algorithm 28, minimum phase, spectrally selective, and spatially nonselective RF pulses were designed to excite either ${\text{HCO}}_{3}^{-}$ (160 ppm, 20° flip angle) or CO_2_ (124.5 ppm, 70° flip angle) individually, while leaving [1‐^13^C]pyruvate (170 ppm) unexcited (parameters: duration 10 ms, time bandwidth product 6, 3.0‐ppm passband (95%), 10^−5^ stopband (6.5 ppm from the center of the passband)). An open‐source RF pulse design toolbox was used to generate these pulses (http://rsl.stanford.edu/research/software.html). The RF pulse for 20° ${\text{HCO}}_{3}^{-}$ excitation is shown in Figure 1a. The corresponding spectral profiles, shown in Figure 1b, are acquired by sweeping the transmitter frequency across a 2000 kHz (26 ppm) range, and obtaining individual free induction decays (FIDs) from a phantom containing thermally polarized, 5M ^13^C enriched urea (phantom T_1_ 75 ms; scan parameters: TR 400 ms, 8 kHz bandwidth, 2048 points, 256 ms readout, nominal flip angle 20° on resonance, 81 frequency steps). The experimental profiles show good agreement with Bloch simulation, revealing a suppression level of at least 10^−3^, with the remaining discrepancies likely caused by RF amplifier nonlinearities 29.

An ECG‐gated pulse‐and‐acquire sequence was used to demonstrate the signal‐to‐noise ratio (SNR) gain from the large flip angle excitation and to determine the optimal timing window for subsequent imaging experiments of myocardial ${\text{HCO}}_{3}^{-}$ and CO_2_ (parameters: TR 1 s, 13 kHz bandwidth, 8192 points, 630 ms readout). The sequence alternated between excitation of all metabolites (hard pulse, 50 μs duration, 10°), of ${\text{HCO}}_{3}^{-}$ only (10 ms duration, 20°), or of CO_2_ only (10 ms duration, 70°).

**Figure 2:**
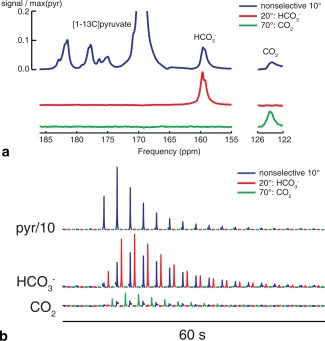
(a) Summed spectra obtained following injection of [1‐^13^C]pyruvate are shown using different excitation schemes (blue: nonselective; red: ${\text{HCO}}_{3}^{-}$ only; green: CO_2_ only). (b) Time courses over 1 min of data acquisition, showing the SNR gain from selective, large flip angle excitation.

Figure 2a shows the sum of 1 min of data acquisition obtained following injection of hyperpolarized [1‐^13^C]pyruvate using the different excitation pulses. These spectra show signals from a nonselective 10° hard pulse excitation in blue, as well as signals from spectrally selective excitation (20° centered on ${\text{HCO}}_{3}^{-}$ in red, 70° centered on CO_2_ in green). The acquired spectra are localized to the heart as a result of coil placement. Figure 2b shows 1 min of dynamic ECG‐gated spectra using the alternating excitation scheme. It is clear that improved signal‐to‐noise ratios are obtained by using the selective excitation pulse. The higher flip angle selective pulses result in an SNR gain of 1.7‐fold for ${\text{HCO}}_{3}^{-}$ and 3.1‐fold for CO_2_. Furthermore, despite the large flip angles (70°) delivered to the CO_2_ resonance, rapid exchange with the ${\text{HCO}}_{3}^{-}$ pool and new production from unaffected pyruvate regenerate this peak for subsequent measurements.

### 3D Imaging Pulse Sequence

**Figure 3:**
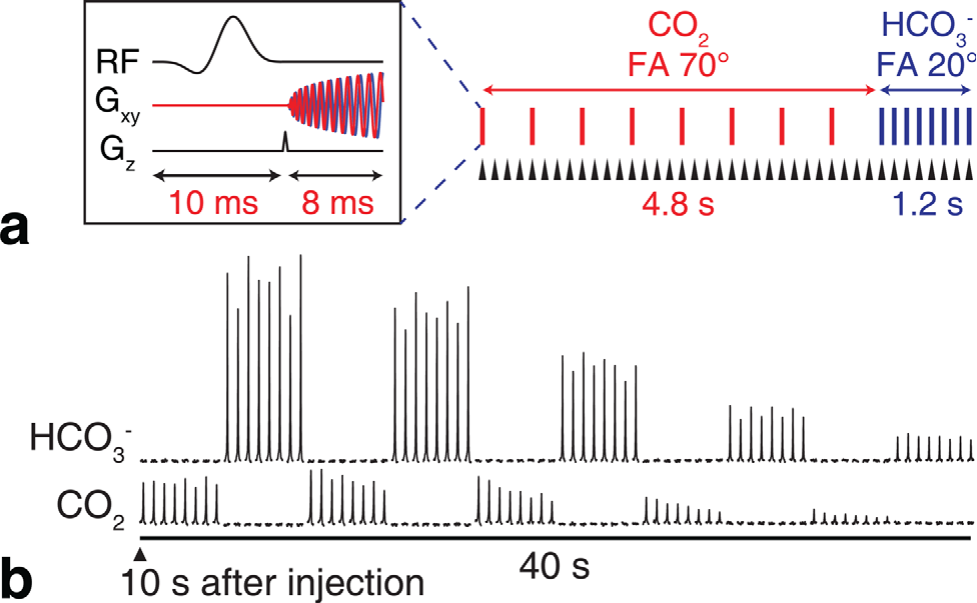
(a) 3D sequence used to image ${\text{HCO}}_{3}^{-}$ and CO_2_ in the heart. Eight phase encodes are required per volume; the TR is varied to allow magnetization transfer between CO_2_ and ${\text{HCO}}_{3}^{-}$ pools following large flip angle excitation of CO_2_. Each triangular arrowhead indicates the beginning of a cardiac RR interval. (b) Nonlocalized ECG‐gated spectra show the feasibility of this approach.

Figure 3a shows the 3D ECG‐gated pulse sequence used to obtain axial images of ${\text{HCO}}_{3}^{-}$ and CO_2_ in the heart. A stack‐of‐spirals trajectory was used for spatial encoding (FOV 60 × 60 × 40 mm^3^, readout duration 6 ms, in‐plane resolution 4.5 × 4.5 × 5.0 mm^2^, 8 phase encodes, TE 4.3 ms). The eight phase encodes were acquired in a linear order across k‐space. Sequential CO_2_ and ${\text{HCO}}_{3}^{-}$ volumes were acquired by switching the excitation pulse used in each block. The TR was varied between the two resonances to allow for regeneration of CO_2_ from the larger ${\text{HCO}}_{3}^{-}$ resonance (TR 500 ms for CO_2_, 150 ms for ${\text{HCO}}_{3}^{-}$). The time required to acquire the two metabolic volumes was 6 s.

Figure 3b shows the feasibility of using this acquisition scheme for 3D imaging. Whole‐heart ECG‐gated spectra were acquired with the readout gradients turned off with the repetition time set to 500 ms for both resonances. The scan was started 10 s after the start of injection. This scan timing was determined from the time course in Figure 2b and was chosen to capture both maximum ${\text{HCO}}_{3}^{-}$ and CO_2_ signal. High SNR spectra can be obtained for at least 40 s after the start of the scan.

### Image Reconstruction and Data Analysis

The spiral trajectory was predicted using a premeasured gradient impulse response function 30. Following Fourier transformation (FFT) along the slab direction, non‐Cartesian k‐space samples were gridded using the nonuniform fast Fourier transform 31, 32. Images were corrected for variable flip angle by dividing each image by sin⁡θ. Linear nearest‐neighbor interpolation using the image magnitude was used to fill in missing ${\text{HCO}}_{3}^{-}$ and CO_2_ images in the corresponding time series. pH_i_ was estimated using the Henderson‐Hasselbalch equation ( $\text{pH}={\mathrm{pK}}_{a}+\;\log_{10}{\mathrm{HCO}}_{3}^{‐}/{\mathrm{CO}}_{2}$) with pK_a_ = 6.15. pH_i_ maps were masked by including only voxels that reached four standard deviations above the mean magnitude ${\text{HCO}}_{3}^{-}$ noise signal in the final analysis.

A paired, two‐tailed Student's t‐test was used to compare pH values between rest and dobutamine stress conditions. Statistical significance was considered at the *P* < 0.05 level.

## RESULTS

**Figure 4:**
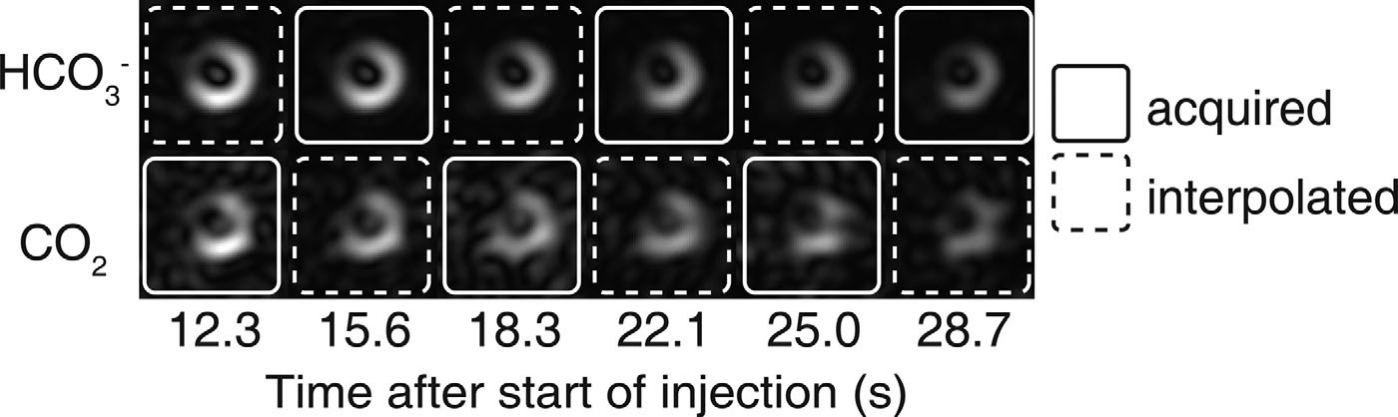
Dynamic imaging of ${\text{HCO}}_{3}^{-}$ and CO_2_ in a midventricular slice following injection of HP [1‐^13^C]pyruvate. The images marked with dashed borders are interpolated from adjacent frames. Images are cropped to a 30 x 30 mm^2^ FOV.

Figure 4 shows dynamic imaging of ${\text{HCO}}_{3}^{-}$ and CO_2_ using the 3D pH_i_ mapping pulse sequence in a midventricular slice. This set of images directly demonstrates the localization of ${\text{HCO}}_{3}^{-}$ and CO_2_ signals to the myocardium, with no detectable signal within the blood pool. The images with dashed borders were linearly interpolated from neighboring time points. The initial missing ${\text{HCO}}_{3}^{-}$ time point was linearly extrapolated from the first two frames of the ${\text{HCO}}_{3}^{-}$ time series. The acquisition time for the central k_z_ spiral readout was used as the time point for each image, and this varied between frames resulting from slight variations in heart rate over the course of the scan.

**Figure 5:**
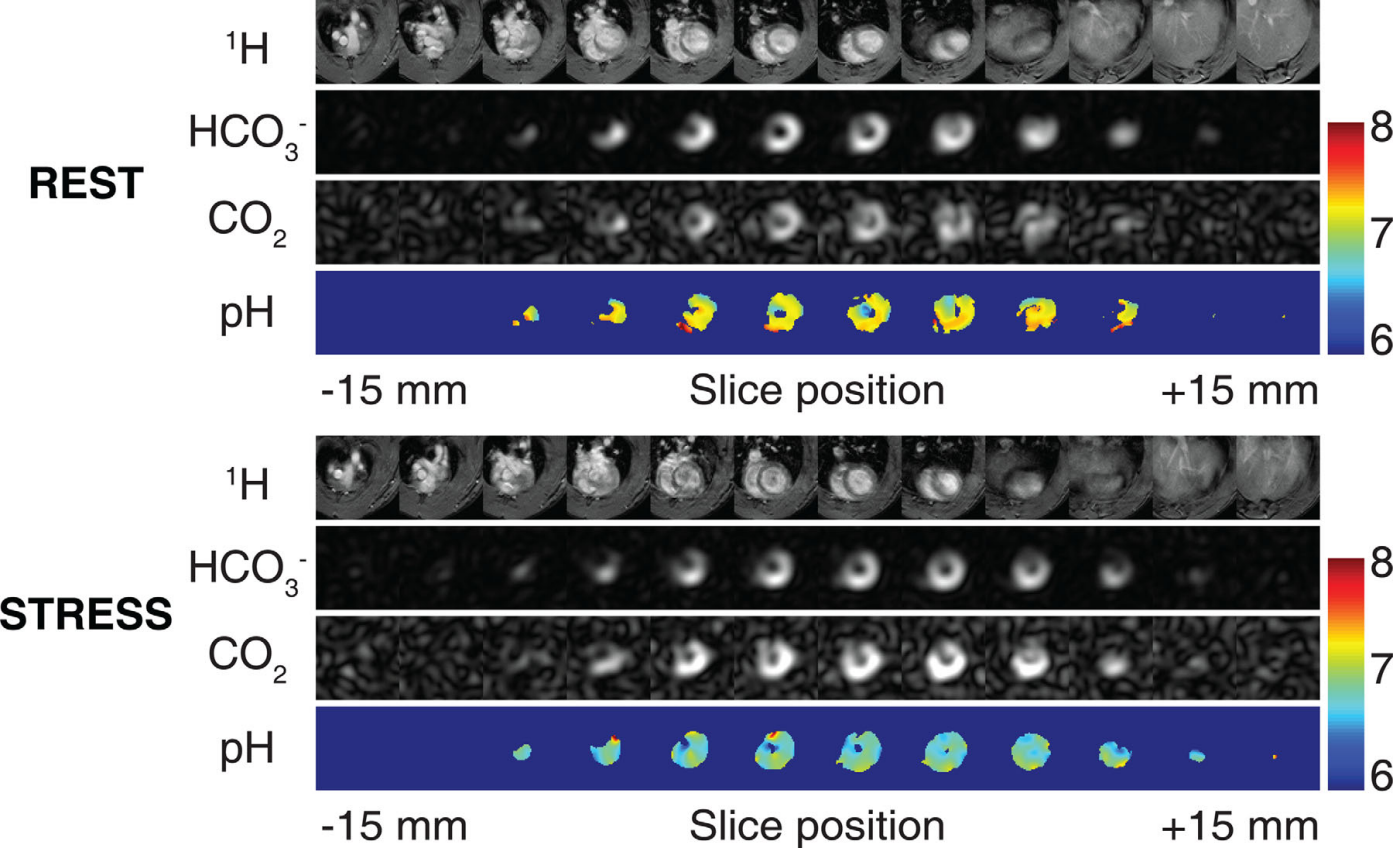
Volumetric images of ${\text{HCO}}_{3}^{-}$ and CO_2_ using the 3D sequence. The first 20 s of data are summed. CO_2_ intensity is increased 10‐fold. Spatial pH_i_ maps reveal pH_i_ = 7.19 ± 0.21 at rest and 6.84 ± 0.17 at stress in this subject. Images are cropped to a 30 x 30 x 30 mm^3^ FOV.

Figure 5 shows volumetric images of ${\text{HCO}}_{3}^{-}$ and CO_2_ in the rodent heart, summed over the first 20 s of data acquisition at both rest and during high cardiac workload. The surface coil placement on the chest wall next to the heart and high cardiac PDH flux localize ${\text{HCO}}_{3}^{-}$ and CO_2_ signals to the heart, demonstrating that any aliasing of ${\text{HCO}}_{3}^{-}$ or CO_2_ from outside of the FOV is negligible. Spatial pH_i_ maps using the ratio between ${\text{HCO}}_{3}^{-}$ and CO_2_ images 20 s following the start of injection show pH_i_ = 7.2 ± 0.2 at rest (mean ± SD) across the heart in this subject. In a separate experiment, injection of hyperpolarized [1‐^13^C]pyruvate during a continuous dobutamine infusion revealed a decrease in pH_i_ to 6.8 ± 0.2 in this subject.

**Figure 6:**
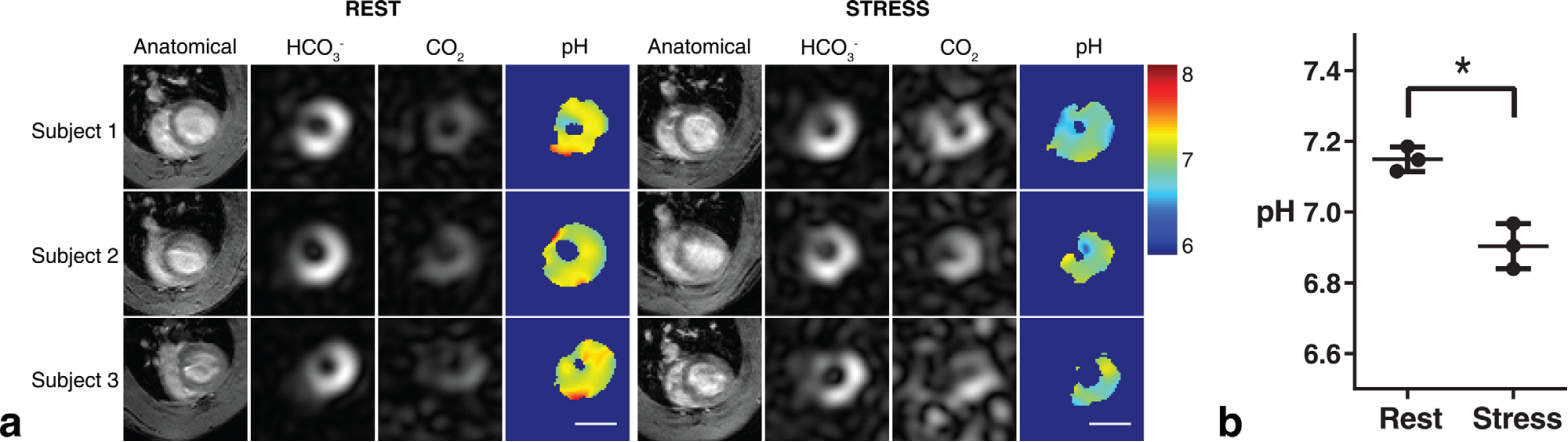
(a) Intracellular pH measurements obtained using the ratio between ${\text{HCO}}_{3}^{-}$ and CO_2_ from a mid‐ventricular slice. Images are cropped to a 30x30 mm^2^ FOV. The scale bar indicates 1 cm. The ^13^C images are taken from a single time point, 20 seconds after the start of injection. (b) Increased cardiac workload results in decreased apparent pH_i_ in the healthy heart.

Figure 6 shows midventricular pH_i_ maps (20 s after the start of injection) in multiple subjects. The ${\text{HCO}}_{3}^{-}$ and CO_2_ images are localized to the myocardial tissue, as shown by the anatomical segmented GRE images. The pH maps demonstrate a uniform myocardial pH_i_ of 7.15 ± 0.04 (mean ± SD over the group, n = 3) across the whole heart, at rest, consistent with global pH_i_ measurements in rats and pigs 18, 25. Following 15 min of high cardiac workload, pH_i_ decreased to 6.90 ± 0.06 (*P* < 0.05, paired t‐test). Figure 6b shows the statistical comparison between mean myocardial pH_i_ at rest and during dobutamine stress.

## DISCUSSION

### Significance

The proper functioning of cells relies on tight control of intracellular pH. Despite the vital importance of this physiological parameter, rapid noninvasive mapping of intracellular pH within the in vivo heart is impossible with existing techniques. Here, we build upon the previous use of hyperpolarized substrates 18, 19, 25 and describe a new method that exploits the body's natural ${\text{HCO}}_{3}^{-}$/CO_2_ buffering system to obtain a spatial three‐dimensional map of the myocardial intracellular pH. The sensitivity of the method is demonstrated by detection of a decrease in pH_i_ following 15 min of high cardiac workload.

It is anticipated that this development will enable new experimental paradigms in which acute, focal alterations in pH can be detected in vivo. Disruption to the acid‐base balance within cells affects enzyme activity, ion channel behavior, Ca^2+^ handling, and contractile function. Under certain conditions, a decline in cardiac pH_i_ can trigger electrical arrhythmia 2, leading to sudden cardiac death. The ability to create a spatial map of pH_i_ could allow assessment of stress‐inducible ischemia and potential ventricular arrythmogenic substrates within the ischemic heart.

### Technical Discussion

The correct use of the Henderson‐Hasselbalch equation for intracellular pH measurement requires that two necessary conditions are satisfied 25. The first of these conditions is that the hyperpolarized acid‐base pair must be in rapid exchange. This condition is satisfied at physiological pH values in which CA enzyme activity is high. If CA activity is low, as may be the case at reduced pH found in ischemia, then ^13^CO_2_ and H^13^ ${\text{CO}}_{3}^{-}$ may not equilibrate on the time scale of the hyperpolarized ^13^C experiment. The effect of this incomplete equilibration would result in an artefactual low pH_i_ measurement. Previous work has shown agreement between ^13^C and ^31^P‐MRS measurements in isolated, perfused hearts subjected to ischemia and subsequent reperfusion 25, which indicates that CA activity is sufficiently high even following an ischemic event.

The second condition is that the acid‐base pair are detected from the same cellular compartment. In rodents, mitochondrial CA activity is low, and mitochondrial CO_2_ is vented into a cytoplasmic domain with elevated CA activity 24. CO_2_ efflux from the myocardium into the extracellular space is likely low on the experimental timescale, as the ratio between ${\text{HCO}}_{3}^{-}$ and CO_2_ reaches a plateau within 5 s in vivo 25. The images indicate that ${\text{HCO}}_{3}^{-}$ and CO_2_ are confined to the myocardium, which is dominated by the intracellular space. The close agreement of the hyperpolarized ^13^C pH measurement with the ^31^P measurement in the perfused heart indicates minimal contamination with the extracellular space 25. CO_2_ venting from the myocardium into the blood is likely low, as no ${\text{HCO}}_{3}^{-}$ or CO_2_ signals were visualized within the blood pool here. The absence of any ${\text{HCO}}_{3}^{-}$ and CO_2_ signal within the blood pool is consistent with dilution into a much larger volume, the fact that mature erythrocytes are anucleate and lack mitochondria, and association of ^13^CO_2_ with hemoglobin, which would shorten the T_1_ and rapidly remove the hyperpolarized signal. It is interesting to contrast the rapid steady state reached in rodents to measurements made in pigs, in which the measured pH_i_ appears to rise over a 20 s period, suggesting a difference between species in the rate at which ^13^CO_2_ leaves the cytosolic compartment during the duration of the experiment 18.

Dobutamine, a β_1_‐adrenergic agonist, is a positive inotrope used clinically to increase cardiac workload as a pharmacologic stress testing agent 33. Continuous dobutamine infusion and increased cardiac workload result in increased PDH and TCA cycle flux, as well as increased glycolysis, leading to the production of excess intracellular lactic acid 34, 35. The delivery of hyperpolarized pyruvate directly probes an increase in the intracellular lactate pool size, as demonstrated by previous studies using hyperpolarized pyruvate in rats 36, 37 and pigs 38, 39. Here, we demonstrate that the increase in glycolysis results in an appreciable intracellular pH change during normoxia, which can also be probed using hyperpolarized [1‐^13^C]pyruvate, despite the high intracellular buffering capacity and normal perfusion of the myocardium. Similarly, increased cardiac workload reduces intracellular pH in isolated cardiomyocytes during high‐frequency electrical stimulation 40, as well as following β‐adrenergic stimulation using isoprenaline 41.

The optimized acquisition in this study uses spectrally selective imaging of the ${\text{HCO}}_{3}^{-}$ and CO_2_ resonances to preserve the limited reservoir of hyperpolarized [1‐^13^C]pyruvate magnetization 18, 42, 43, 44, 45, 46, 47. As the method is based on the ratio between ${\text{HCO}}_{3}^{-}$ and CO_2_ signals, the measured pH is independent of the concentration of pyruvate or its downstream metabolites. The main requirement is that the PDH flux is high enough to enable the detection of CO_2_ and ${\text{HCO}}_{3}^{-}$ within the cell. One limitation of this approach is that the arrival of pyruvate into the heart is not monitored. Although pyruvate concentration is not required for pH determination, imaging the arrival of hyperpolarized pyruvate at the heart would allow a simultaneous measurement of cardiac PDH flux, which would be useful to assess tissue viability 18. Pyruvate images could also be used to determine the optimal time to acquire ${\text{HCO}}_{3}^{-}$ and CO_2_ images, which would be useful in a heterogeneous subject group in which the arterial input function is expected to be variable. Alternatively, co‐polarization of metabolically inert ^13^C urea alongside [1‐^13^C]pyruvate could be used to obtain the arrival time without disturbing the hyperpolarized pyruvate magnetization 48, 49.

The limiting factor for accurate pH_i_ determination is the relatively low SNR of the CO_2_ signal specifically, which is 10% of the ${\text{HCO}}_{3}^{-}$ signal at physiological pH levels. This is consistent with an error propagation analysis of the Henderson‐Hasselbalch equation, which leads to $\sigma_{pH}^{2}={\left(1/{\mathrm{SNR}}_{\mathrm{bic}}\right)}^{2}+{\left(1/{\mathrm{SNR}}_{\mathrm{CO2}}\right)}^{2}$, where $\sigma_{X}$ denotes the standard deviation of X, and ${\mathrm{SNR}}_{X}=X/\sigma_{X}$ denotes the signal‐to‐noise ratio. The SNR of the CO_2_ is typically ∼5 in the in vivo experiments shown here, which is consistent with the single subject standard deviation reported in Figure 5. A 70° flip angle was used to excite CO_2_; this angle was chosen to improve SNR while avoiding excitation by flip angles above 90° due to $B_{1}^{+}$ inhomogeneity, which would decrease the CO_2_ signal. This is a reasonable trade‐off, as the transverse magnetization created by 70° excitations is 94% that of a 90° excitation.

The CO_2_ SNR may further be improved by shortening the duration of the CO_2_ excitation. Specifically, the 10 ms pulse used for excitation of ${\text{HCO}}_{3}^{-}$ (170 ppm) was designed to avoid excitation of [1‐^13^C]pyruvate (160 ppm). CO_2_, which is positioned at 124.5 ppm, is three times further away in chemical shift, and a shortened pulse (∼3.5 ms) would reduce the effective echo time and reduce $T_{2}^{*}$‐related dephasing across the volume. Although this could potentially improve SNR, the difference in echo time would need to be compensated by $T_{2}^{*}$ mapping within the heart. This could be done using multiecho ^1^H images, and converting the $T_{2}^{*}$ values to ^13^C via multiplication by γ_1H_/γ_13C_ ≈ 4.

Currently, the validation of noninvasive methods for intracellular pH mapping within the myocardium is challenging. The gold standard for noninvasive intracellular pH measurement, ^31^P‐MRS, suffers from contamination of the pH‐sensitive P_i_ resonance with 2,3‐diphosphoglycerate (2,3‐DPG) found in the ventricular blood. It is possible to perform a saturation transfer experiment to remove the contribution from 2,3‐DPG, but this method remains limited to a whole‐heart measurement with long scan time due to limited SNR 8. The ^13^C and ^31^P‐based measurements agree in isolated, perfused rat hearts in which blood is not present 25. Invasive measurements using either blood replacement 6 or open‐chest techniques 7 may be of value for correlation with the noninvasive hyperpolarized ^13^C measurements described here.

### Clinical Translation

The use of hyperpolarized ^13^C substrates to study human heart disease is rapidly approaching the clinic. The first application of hyperpolarized magnetic resonance in humans at the University of California, San Francisco, demonstrated the potential of the technique to stage prostate cancer and assess the response to treatment 50. New hyperpolarizer designs that produce sterile, clinical‐grade tracers for human use are now available 51. Hyperpolarized pyruvate is approved for use in physiological studies that will be centered on assessing metabolic conversion within the heart into lactate, alanine, and bicarbonate as sensitive markers of cellular viability and metabolic state. Therefore, intracellular pH mapping, using the metabolic products of hyperpolarized [1‐^13^C]pyruvate, has a direct path for clinical translation.

## CONCLUSIONS

An optimized sequence for mapping intracellular pH within the rodent heart in under a minute is proposed. Following an injection of hyperpolarized [1‐^13^C]pyruvate, mitochondrial decarboxylation results in cytosolic CO_2_ and ${\text{HCO}}_{3}^{-}$. Spectrally selective excitation is combined with a rapid 3D imaging readout, and the rapid regeneration of CO_2_ due to carbonic anhydrase‐mediated exchange is used to obtain whole‐heart intracellular pH maps in vivo. The intracellular pH was measured to be 7.15 ± 0.04 at baseline, and decreased to 6.90 ± 0.06 following 15 min of continuous β‐adrenergic stimulation. The new method is anticipated to enable assessment of stress‐inducible ischemia and potential ventricular arrhythmogenic substrates within the ischemic heart.

## References

[1] Vaughan‐Jones RD , Spitzer KW , Swietach P. Intracellular pH regulation in heart. J Mol Cell Cardiol 2009;46:318–331. 19041875 10.1016/j.yjmcc.2008.10.024

[2] Orchard CH , Cingolani HE. Acidosis and arrhythmias in cardiac muscle. Cardiovasc Res 1994;28:1312–1319. 7954638 10.1093/cvr/28.9.1312

[3] Frohlich O , Wallert MA. Methods of measuring intracellular pH in the heart. Cardiovasc Res 1995;29:194–202. 7736495 10.1016/s0008-6363(96)88570-1

[4] Hoult DI , Busby SJ , Gadian DG , Radda GK , Richards RE , Seeley PJ. Observation of tissue metabolites using 31P nuclear magnetic resonance. Nature 1974;252:285–287. 4431445 10.1038/252285a0

[5] Bailey IA , Williams SR , Radda GK , Gadian DG. Activity of phosphorylase in total global ischaemia in the rat heart. A phosphorus‐31 nuclear‐magnetic‐resonance study. Biochem J 1981;196:171–178. 7306067 10.1042/bj1960171 PMC1162979

[6] Katz LA , Swain JA , Portman MA , Balaban RS. Intracellular pH and inorganic phosphate content of heart in vivo: a 31P‐NMR study. Am J Physiol 1988;255:H189–H196. 3394819 10.1152/ajpheart.1988.255.1.H189

[7] Brindle KM , Rajagopalan B , Williams DS , Detre JA , Simplaceanu E , Ho C , Radda GK. 31P NMR measurements of myocardial pH in vivo. Biochem Biophys Res Comm 1988;151:70–77. 3348798 10.1016/0006-291x(88)90560-8

[8] Blamire AM , Rajagopalan B , Radda GK. Measurement of myocardial pH by saturation transfer in man. Magn Reson Med 1999;41:198–203. 10025630 10.1002/(sici)1522-2594(199901)41:1<198::aid-mrm28>3.0.co;2-h

[9] van Sluis R , Bhujwalla ZM , Raghunand N , Ballesteros P , Alvarez J , Cerdan S , Galons JP , Gillies RJ. In vivo imaging of extracellular pH using 1H MRSI. Magn Reson Med 1999;41:743–750. 10332850 10.1002/(sici)1522-2594(199904)41:4<743::aid-mrm13>3.0.co;2-z

[10] Vermathen P , Capizzano AA , Maudsley AA. Administration and (1)H MRS detection of histidine in human brain: application to in vivo pH measurement. Magn Reson Med 2000;43:665–675. 10800031 10.1002/(sici)1522-2594(200005)43:5<665::aid-mrm8>3.0.co;2-3

[11] Gillies RJ , Liu Z , Bhujwalla Z. 31P‐MRS measurements of extracellular pH of tumors using 3‐aminopropylphosphonate. Am J Physiol 1994;267:C195–C203. 8048479 10.1152/ajpcell.1994.267.1.C195

[12] Mason RP. Transmembrane pH gradients in vivo: measurements using fluorinated vitamin B6 derivatives. Curr Med Chem 1999;6:481–499. 10213795

[13] Ward KM , Balaban RS. Determination of pH using water protons and chemical exchange dependent saturation transfer (CEST). Magn Reson Med 2000;44:799–802. 11064415 10.1002/1522-2594(200011)44:5<799::aid-mrm18>3.0.co;2-s

[14] Zhou J , Payen JF , Wilson DA , Traystman RJ , van Zijl PC. Using the amide proton signals of intracellular proteins and peptides to detect pH effects in MRI. Nat Med 2003;9:1085–1090. 12872167 10.1038/nm907

[15] Ardenkjaer‐Larsen JH , Fridlund B , Gram A , Hansson G , Hansson L , Lerche MH , Servin R , Thaning M , Golman K. Increase in signal‐to‐noise ratio of > 10,000 times in liquid‐state NMR. Proc Natl Acad Sci U S A 2003;100:10158–10163. 12930897 10.1073/pnas.1733835100 PMC193532

[16] Golman K , Petersson JS , Magnusson P , Johansson E , Akeson P , Chai C‐M , Hansson G , Månsson S. Cardiac metabolism measured noninvasively by hyperpolarized 13C MRI. Magn Reson Med 2008;59:1005–1013. 18429038 10.1002/mrm.21460

[17] Schroeder MA , Cochlin LE , Heather LC , Clarke K , Radda GK , Tyler DJ. In vivo assessment of pyruvate dehydrogenase flux in the heart using hyperpolarized carbon‐13 magnetic resonance. Proc Natl Acad Sci U S A 2008;105:12051–12056. 18689683 10.1073/pnas.0805953105 PMC2515222

[18] Chen AP , Hurd RE , Schroeder MA , Lau AZ , Gu YP , Lam WW , Barry J , Tropp J , Cunningham CH. Simultaneous investigation of cardiac pyruvate dehydrogenase flux, Krebs cycle metabolism and pH, using hyperpolarized [1,2‐(13)C2]pyruvate in vivo. NMR Biomed 2012;25:305–311. 21774012 10.1002/nbm.1749 PMC4618301

[19] Gallagher FA , Kettunen MI , Day SE , et al. Magnetic resonance imaging of pH in vivo using hyperpolarized 13C‐labelled bicarbonate. Nature 2008;453:940–943. 18509335 10.1038/nature07017

[20] Scholz DJ , Janich MA , Kollisch U , Schulte RF , Ardenkjaer‐Larsen JH , Frank A , Haase A , Schwaiger M , Menzel MI. Quantified pH imaging with hyperpolarized (13) C‐bicarbonate. Magn Reson Med 2015;73:2274–2282. 25046867 10.1002/mrm.25357

[21] Scholz DJ , Otto AM , Hintermair J , et al. Parameterization of hyperpolarized (13)C‐bicarbonate‐dissolution dynamic nuclear polarization. MAGMA 2015;28:591–598. 26449715 10.1007/s10334-015-0500-9

[22] Korenchan DE , Flavell RR , Baligand C , Sriram R , Neumann K , Sukumar S , VanBrocklin H , Vigneron DB , Wilson DM , Kurhanewicz J. Dynamic nuclear polarization of biocompatible (13)C‐enriched carbonates for in vivo pH imaging. Chem Commun (Camb) 2016;52:3030–3033. 26792559 10.1039/c5cc09724j PMC4864526

[23] Flavell RR , von Morze C , Blecha JE , et al. Application of Good's buffers to pH imaging using hyperpolarized (13)C MRI. Chem Commun (Camb) 2015;51:14119–14122. 26257040 10.1039/c5cc05348j PMC4854286

[24] Schroeder MA , Ali MA , Hulikova A , Supuran CT , Clarke K , Vaughan‐Jones RD , Tyler DJ , Swietach P. Extramitochondrial domain rich in carbonic anhydrase activity improves myocardial energetics. Proc Natl Acad Sci U S A 2013;110:E958–E967. 23431149 10.1073/pnas.1213471110 PMC3593844

[25] Schroeder MA , Swietach P , Atherton HJ , Gallagher FA , Lee P , Radda GK , Clarke K , Tyler DJ. Measuring intracellular pH in the heart using hyperpolarized carbon dioxide and bicarbonate: a 13C and 31P magnetic resonance spectroscopy study. Cardiovasc Res 2010;86:82–91. 20008827 10.1093/cvr/cvp396 PMC2836261

[26] Cassidy PJ , Schneider JE , Grieve SM , Lygate CA , Tyler DJ , Neubauer S , Clarke K. An animal handling system for small animals in vivo MR. Proc ISMRM 2005;13:488.

[27] Schneider JE , Barnes H , Neubauer S , Jezzard P. Automated‐shim approach to facilitate 1H‐MRS in mouse hearts in vivo. In Proceedings of the 17th Annual Meeting of ISMRM, Honolulu, Hawaii, USA, 2009. Abstract 1782.

[28] Pauly J , Le Roux P , Nishimura D , Macovski A. Parameter relations for the Shinnar‐Le Roux selective excitation pulse design algorithm [NMR imaging]. IEEE Trans Med Imaging 1991;10:53–65. 18222800 10.1109/42.75611

[29] Chan F , Pauly J , Macovski A. Effects of RF amplifier distortion on selective excitation and their correction by prewarping. Magn Reson Med 1992;23:224–238. 1549038 10.1002/mrm.1910230204

[30] Vannesjo SJ , Haeberlin M , Kasper L , Pavan M , Wilm BJ , Barmet C , Pruessmann KP. Gradient system characterization by impulse response measurements with a dynamic field camera. Magn Reson Med 2013;69:583–593. 22499483 10.1002/mrm.24263

[31] Fessler JA , Sutton BP. Nonuniform fast Fourier transforms using min‐max interpolation. IEEE Trans Sig Process 2003;51:560–574.

[32] Zwart NR , Johnson KO , Pipe JG. Efficient sample density estimation by combining gridding and an optimized kernel. Magn Reson Med 2012;67:701–710. 21688320 10.1002/mrm.23041

[33] Tuttle RR , Mills J. Dobutamine: development of a new catecholamine to selectively increase cardiac contractility. Circ Res 1975;36:185–196. 234805 10.1161/01.res.36.1.185

[34] Sharma N , Okere IC , Brunengraber DZ , McElfresh TA , King KL , Sterk JP , Huang H , Chandler MP , Stanley WC. Regulation of pyruvate dehydrogenase activity and citric acid cycle intermediates during high cardiac power generation. J Physiol 2005;562:593–603. 15550462 10.1113/jphysiol.2004.075713 PMC1665507

[35] O'Donnell JM , Kudej RK , LaNoue KF , Vatner SF , Lewandowski ED. Limited transfer of cytosolic NADH into mitochondria at high cardiac workload. Am J Physiol Heart Circ Physiol 2004;286:H2237–H2242. 14751856 10.1152/ajpheart.01113.2003

[36] Schroeder MA , Atherton HJ , Dodd MS , Lee P , Cochlin LE , Radda GK , Clarke K , Tyler DJ. The cycling of acetyl‐coenzyme A through acetylcarnitine buffers cardiac substrate supply: a hyperpolarized 13C magnetic resonance study. Circ Cardiovasc Imaging 2012;5:201–209. 22238215 10.1161/CIRCIMAGING.111.969451 PMC3378498

[37] Josan S , Park JM , Hurd R , Yen YF , Pfefferbaum A , Spielman D , Mayer D. In vivo investigation of cardiac metabolism in the rat using MRS of hyperpolarized [1‐13C] and [2‐13C]pyruvate. NMR Biomed 2013;26:1680–1687. 23904148 10.1002/nbm.3003 PMC3838505

[38] Menichetti L , Frijia F , Flori A , et al. Assessment of real‐time myocardial uptake and enzymatic conversion of hyperpolarized [1‐(13) C]pyruvate in pigs using slice selective magnetic resonance spectroscopy. Contrast Media Mol Imaging 2012;7:85–94. 22344884 10.1002/cmmi.480

[39] Chen AP , Lau AZ , Gu Y , Schroeder MA , Barry J , Cunningham CH. Investigation of cardiac malate‐aspartate shuttle at high workload using hyperpolarized [1,2‐^13^C_2_]pyruvate. In Proceedings of the 21st Annual Meeting of ISMRM, Salt Lake City, Utah, USA, 2013. Abstract 4511.

[40] Bountra C , Kaila K , Vaughan‐Jones RD. Effect of repetitive activity upon intracellular pH, sodium and contraction in sheep cardiac Purkinje fibres. J Physiol 1988;398:341–360. 3392677 10.1113/jphysiol.1988.sp017046 PMC1191776

[41] Shida S , Nakaya H , Matsumoto S , Kanno M. Beta 1 adrenoceptor mediated decrease in pHi in quiescent ventricular myocardium. Cardiovasc Res 1994;28:112–118. 7906613 10.1093/cvr/28.1.112

[42] Chen WC , Teo XQ , Lee MY , Radda GK , Lee P. Robust hyperpolarized (13)C metabolic imaging with selective non‐excitation of pyruvate (SNEP). NMR Biomed 2015;28:1021–1030. 26119950 10.1002/nbm.3346

[43] Chen AP , Cunningham CH. Single voxel localization for dynamic hyperpolarized (13)C MR spectroscopy. J Magn Reson 2015;258:81–85. 26232365 10.1016/j.jmr.2015.07.002

[44] Lau AZ , Chen AP , Ghugre NR , Ramanan V , Lam WW , Connelly KA , Wright GA , Cunningham CH. Rapid multislice imaging of hyperpolarized (13)C pyruvate and bicarbonate in the heart. Magn Reson Med 2010;64:1323–1331. 20574989 10.1002/mrm.22525

[45] Lau AZ , Chen AP , Hurd RE , Cunningham CH. Spectral‐spatial excitation for rapid imaging of DNP compounds. NMR Biomed 2011;24:988–996. 21751271 10.1002/nbm.1743

[46] Miller JJ , Lau AZ , Teh I , Schneider JE , Kinchesh P , Smart S , Ball V , Sibson NR , Tyler DJ. Robust and high resolution hyperpolarized metabolic imaging of the rat heart at 7 T with 3D spectral‐spatial EPI. Magn Reson Med 2016;75:1515–1524. 25991606 10.1002/mrm.25730 PMC4556070

[47] Larson PEZ , Kerr AB , Chen AP , Lustig MS , Zierhut ML , Hu S , Cunningham CH , Pauly JM , Kurhanewicz J , Vigneron DB. Multiband excitation pulses for hyperpolarized 13C dynamic chemical‐shift imaging. J Magn Reson 2008;194:121–127. 18619875 10.1016/j.jmr.2008.06.010 PMC3739981

[48] Lau AZ , Miller JJ , Robson MD , Tyler DJ. Cardiac perfusion imaging using hyperpolarized 13C urea using flow sensitizing gradients. Magn Reson Med 2016;75:1474–1483. 25991580 10.1002/mrm.25713 PMC4556069

[49] Lau AZ , Miller JJ , Robson MD , Tyler DJ. Simultaneous assessment of cardiac metabolism and perfusion using copolarized [1‐ C]pyruvate and C‐urea. Magn Reson Med 2017;77:151–158. 10.1002/mrm.26106 PMC5217077 26743440

[50] Nelson SJ , Kurhanewicz J , Vigneron DB , et al. Metabolic imaging of patients with prostate cancer using hyperpolarized [1‐13C]Pyruvate. Sci Transl Med 2013;5:198ra108. 10.1126/scitranslmed.3006070 PMC4201045 23946197

[51] Ardenkjaer‐Larsen JH , Leach AM , Clarke N , Urbahn J , Anderson D , Skloss TW. Dynamic nuclear polarization polarizer for sterile use intent. NMR Biomed 2011;24:927–932. 21416540 10.1002/nbm.1682

